# T-cells in chronic lymphocytic leukemia: Guardians or drivers of disease?

**DOI:** 10.1038/s41375-020-0873-2

**Published:** 2020-05-26

**Authors:** Philipp M. Roessner, Martina Seiffert

**Affiliations:** Molecular Genetics, German Cancer Research Center (DKFZ), Heidelberg, Germany

**Keywords:** Cancer microenvironment, Chronic lymphocytic leukaemia

## Abstract

Chronic lymphocytic leukemia (CLL) is a B-cell malignancy, which is associated with profound alterations and defects in the immune system and a prevalent dependency on the microenvironmental niche. An abnormal T-cell compartment in the blood of CLL patients was already reported 40 years ago. Since then, our knowledge of T-cell characteristics in CLL has grown steadily, but the question of whether T-cells act as pro-tumoral bystander cells or possess anti-tumoral activity is still under debate. Increased numbers of CD4^+^ T-helper cell subsets are present in the blood of CLL patients, and T-helper cell cytokines have been shown to stimulate CLL cell survival and proliferation in vitro. In line with this, survival and growth of CLL cells in murine xenograft models have been shown to rely on activated CD4^+^ T-cells. This led to the hypothesis that T-cells are tumor-supportive in CLL. In recent years, evidence for an enrichment of antigen-experienced CD8^+^ T-cells in CLL has accumulated, and these cells have been shown to control leukemia in a CLL mouse model. Based on this, it was suggested that CD8^+^ T-cells recognize CLL-specific antigens and exert an anti-leukemia function. As described for other cancer entities, T-cells in CLL express multiple inhibitory receptors, such as PD-1, and lose their functional capacity, leading to an exhaustion phenotype which has been shown to be more severe in T-cells from secondary lymphoid organs compared with peripheral blood. This exhausted phenotype has been suggested to be causative for the poor response of CLL patients to CAR T-cell therapies. In addition, T-cells have been shown to be affected by drugs that are used to treat CLL, which likely impacts therapy response. This review provides an overview of the current knowledge about alterations of T-cells in CLL, including their distribution, function, and exhaustion state in blood and lymphoid organs, and touches also on the topic of how CLL drugs impact on the T-cell compartment and recent results of T-cell-based immunotherapy. We will discuss potential pathological roles of T-cell subsets in CLL and address the question of whether they foster progression or control of disease.

## CD4^+^ T-cells

**Fig. 1:**
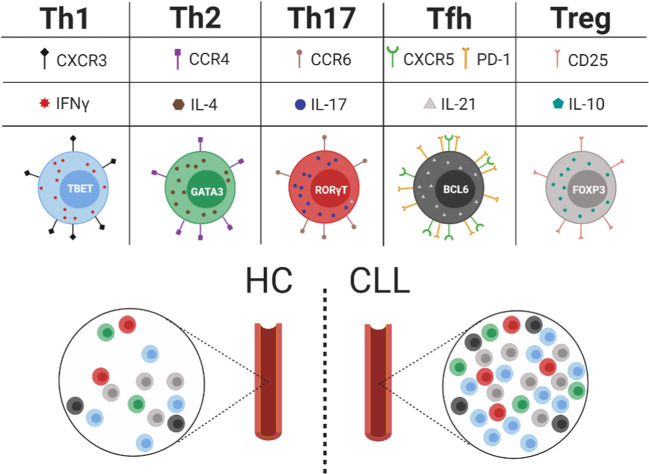
CD4^+^ T-cell subset diversity and distribution in CLL. CD4^+^ T-cell subsets are defined by the expression of distinct surface markers (CXCR3 (2, 3), CCR4 (2), CCR6 (2), CXCR5 (3), PD-1 (3) and CD25 (4)), cytokines (IFNγ (1–3), IL-4 (1–3), IL-17 (1–3), IL-21 (1, 3) and IL-10 (1, 4)), and transcription factors (TBET (3), GATA3 (3), RORγT (3), BCL6 (3) and FOXP3(4)). In comparison to healthy controls (HC), CD4^+^ T-cell subsets are more abundant in the blood of CLL patients.

CD4^+^ T-cells are a heterogeneous population of cells, broadly divided into conventional CD4^+^ (cCD4^+^) T-cells and forkhead box protein P3 (FOXP3^+^) expressing regulatory T-cells (Treg). The identification of cell type-specific cytokines and functions resulted in the definition of three subsets of cCD4^+^ T-helper (Th) cells, namely Th1, Th2, and Th17 T-cells [1, 2]. More recently, follicular helper T-cells (Tfh) have been described as the most common Th T-cell subset of lymphoid organs [3]. An overview of subset-defining surface markers, key cytokines, and transcriptional regulators is provided in Fig. 1.

The majority of CD4^+^ T-cells orchestrate the immune responses of surveilling immune cells, e.g., CD8^+^ T-cells and B-cells, against Th-specific pathogens [1]. In contrast to the T-helper subsets, Tregs are thought to be immunosuppressive and reduce the activity of effector T-cells [4]. Recent investigations identified cytotoxic CD4^+^ T-cells, which kill target cells by granzyme (Gzm)B and perforins in an antigen-specific manner, especially in conditions of chronic viral infections [5].

## CD4^+^ T-cells in CLL

### Phenotype of CD4^+^ T-cells in CLL

An enrichment of antigen-experienced memory and effector CD4^+^ T-cells accompanied by a relative loss of naïve CD4^+^ T-cells has been repeatedly observed in chronic lymphocytic leukemia (CLL) [6–8]. Moreover, CLL-associated CD4^+^ T-cells express higher levels of the inhibitory receptors programmed cell death protein 1 (PD-1) [6, 7, 9–11], CD160 [9], and T-cell immunoreceptor with Ig and ITIM domains (TIGIT) [8] as well as human leukocyte antigen (HLA)-DR as a marker of activation [6]. Ki-67, a marker for cell proliferation, was detected at higher levels in CLL-derived CD4^+^ T-cells in comparison to control T-cells [7]. Hence, CD4^+^ T-cells in the blood of CLL patients seem to be more strongly activated than the T-cells from healthy individuals.

### T-helper cell subsets in CLL

As described above, T-helper cell subsets secrete distinct cytokines and exert specific immunological functions. To understand their role in CLL, the distribution and function of these subsets in patient samples need to be determined. Up until now, such attempts have been largely inconclusive, as both the Th1 and Th2 subsets have been reported as being dominant.

In 2005, Görgün and colleagues investigated T-cell function in CLL. Gene expression profiling (GEP) of sorted T-cells from peripheral blood mononuclear cells (PBMCs) of CLL patients suggested that CD4^+^ T-cells are skewed toward a Th2 phenotype due to decreased expression of *c-Jun NH2-terminal kinase (JNK)* and *p38 mitogen-activated protein kinase (MAPK)* pathway-related genes [12]. This is supported by an increased number of interleukin (IL)-4 secreting CD4^+^ T-cells in PBMCs of CLL patients in comparison to cells of healthy controls (HC). Studies using the immunocompetent Eµ-TCL1 mouse model of CLL and after adoptive transfer of TCL1 leukemia (TCL1 AT) into BL/6 wild-type (WT) mice revealed more IL-4 secretion of CD4^+^ T-cells in comparison to control mice [13, 14]. Similarly, conditioned medium of CLL cells was able to induce secretion of IL-4 in allogeneic mixed lymphocyte reactions (MLR) of healthy individuals, suggesting that soluble factors secreted by CLL cells trigger Th2 T-cell polarization in vitro [15].

In contrast, ex vivo mitogen stimulation of PBMCs using phorbol 12-myristate-13-acetate (PMA)/ionomycin led to higher percentages of interferon (IFN)γ-expressing Th1-like T-cells in CLL patients compared with HC [16, 17], which is in line with data from the TCL1 AT mouse model [17]. Based on surface marker expression data, increased absolute numbers of both Th1 and Th2 T-cells have been reported in the blood of CLL patients in comparison to HC [7, 8]. The increase of Th1 T-cells was more pronounced in patients with progressive disease [7]. Recently, expression analysis of surface marker proteins and the master transcriptional regulators T-box transcription factor 21 (TBET) and GATA binding protein 3 (GATA3) in patient samples and the TCL1 AT mouse model revealed a more severe accumulation of Th1-like T-cells compared with Th2 cells in CLL [17].

Th1-like T-cells were shown to activate autologous CLL cells and induce their proliferation in an antigen- and IFNγ-dependent manner [18, 19]. In contrast, *Tbx21*^*−/−*^ bone marrow chimeric mice, which showed a profound reduction in IFNγ-producing Th1 T-cells compared with WT controls, showed a comparable CLL progression to control mice [17].

Higher numbers and frequencies of IL-17-expressing Th17 T-cells have been reported in the blood of CLL patients compared with HC which is in line with elevated plasma levels of IL-17 in CLL, although numbers of Th17 T-cells were much lower than Th1 and Th2 cells [20, 21].

It was suggested that Tfh T-cells are tumor-supportive in CLL, as IL-21, a cytokine that is highly expressed by these cells, enhanced CLL cell proliferation in vitro [22, 23]. Frequencies of this CD4^+^ T-cell subset were found to be higher in blood and particularly lymph nodes of CLL patients [22–25].

In conclusion, an enrichment of T-helper cell subsets in CLL has been reported by several groups. Reports concerning a higher abundance of Th1 or Th2 T-cells are controversial. To date, functional investigations are only available for Th1 T-cells, which showed that reducing their number in a CLL mouse model has no impact on disease progression.

### Regulatory T-cells in CLL

Increased frequencies and numbers of Tregs in the blood of CLL patients compared with HC have been consistently reported [7, 8, 11, 26–34]. This accumulation was shown to be more pronounced in patients’ lymph nodes [25]. Numbers of Tregs in blood positively correlated with malignant B-cell counts, higher Rai and Binet staging, as well as unfavorable genetics, and served as an independent predictor of time to first treatment [29–32, 35, 36], suggesting a negative impact of Tregs on disease progression. Of note, higher secretion of IL-10 and transforming growth factor (TGF)β of CLL-derived Tregs was observed in comparison to control Tregs [27, 28]. Moreover, Tregs of CLL patients exhibited increased expression of markers for activation and immunosuppression, such as cytotoxic T-lymphocyte associated protein 4 (CTLA-4), which was confirmed in Eµ-TCL1 mice [37] as well as the TCL1 AT mouse model [27, 32, 38–40]. Reducing Tregs by 50% using anti-CD25 antibodies in the TCL1 AT model did not affect CLL progression [32]. However, data are lacking in mouse models with a more efficient depletion of Tregs, e.g., *Foxp3*^*DTR*^ mice, in which Tregs can be depleted by injection of diphteria toxin [41]. Such a model would help to clarify the role of these cells in CLL development and progression.

In summary, an increase of Tregs in blood of CLL patients has been observed on numerous occasions, but more functional analyses are still required to elucidate the role of Tregs and other T-helper cell subsets in CLL.

### Influence of CD4^+^ T-cells on CLL progression

The role of CD4^+^ T-cells in CLL, which accumulate in the blood of patients as the disease progresses [17, 42, 43], is discussed with contention. Treatment of CLL cells with Th T-cell-derived factors, such as IFNγ [44], IL-21 [23], IL-4 [45], CD40L [18, 23, 45], or their combinations, either reduced apoptosis or induced proliferation of CLL cells in vitro (Table 1). In line with this, a pro-leukemic effect has been suggested, as autologous CD4^+^ T-cells but not CD8^+^ T-cells were indispensable for inducing CLL cell proliferation in patient-derived xenograft (PDX) mouse models (Table 1) [18, 46]. Co-culture of CLL cells with autologous CD4^+^ T-cells, which were either depleted of PD-1^+^, TIGIT^+^, or PD-1^+^ TIGIT^+^ double-positive T-cells, revealed a reduced CLL cell viability in all of the depleted co-cultures, suggesting that these T-cell subsets enhance CLL cell survival [8]. Interestingly, TIGIT^+^ CD4^+^ T-cells produced higher amounts of IFNγ and IL-10 in a TIGIT-dependent manner than their TIGIT^-^ counterparts [8], which is in line with the aforementioned beneficial effects of IFNγ on CLL cells in vitro. Correlations of CD4^+^ T-cell counts with patients’ progression-free survival (PFS) revealed lower counts to be associated with increased PFS [47]. Accordingly, a higher proportion of PD-1^+^ HLA-DR^+^ CD4^+^ T-cells, which are enriched in CLL patients, was associated with reduced PFS [6].

**Table 1:** Function of CD4^+^ T-cell subsets in CLL.

CD4^+^ T-cell subset	Functional data - in vitro	Functional data - in vivo
Th1	- Activation and proliferation of CLL cells [18, 19]. - rIFNγ inhibits CLL cell apoptosis [44].	- Activation and proliferation of CLL cells in a PDX xenograft model [18]. - Depletion does not affect CLL progression in TCL1 AT mice [17].
Th2	- rIL-4 induces CLL cell proliferation cooperatively with rIL-21 [22]. - rIL-4 rescues CLL cells from apoptosis [45].	-
Th17	-	-
Tfh	- rIL-21 induces CLL cell proliferation cooperatively with rIL-4 [22]. - rIL-21 induces CLL cell proliferation cooperatively with rCD40L stimulation [23].	-
Treg	-	- Depletion by 50% does not affect CLL progression in TCL1 AT mice [32].

Overview of published functional data of CD4^+^ T-cell subsets and their derived cytokines in vitro and the impact of Th T-cell subsets on CLL progression in vivo.

Analyses of the TCL1 AT mouse model of CLL revealed similar alterations of the CD4^+^ T-cell compartment as reported for CLL patients. A relative loss of naïve CD4^+^ T-cells and an accumulation of antigen-experienced CD4^+^ T-cells, as well as a higher expression of the early activation markers CD69 and PD-1, was noted [10], suggesting that this model is a useful tool to investigate the role of CD4^+^ T-cells in CLL.

In contrast to the pro-tumoral effects of CD4^+^ T-cells described above, an anti-tumoral function of CD4^+^ T-cells was suggested by experiments using the Eµ-TCL1 mouse model of CLL. TCL1 AT into GK5 mice that lack CD4^+^ T-cells resulted in a faster CLL development compared with respective treatment of WT mice [48]. However, CD4^+^ T-cell depletion by injecting CD4-specific antibodies into WT mice that had been transplanted with TCL1 leukemia cells induced no difference in CLL development [49]. These controversial results might be explained by differences in the depletion efficacy of CD4^+^ T-cells in GK5 mice versus antibody-treated mice, which might also result in a difference in the degree of simultaneous depletion of other CD4-expressing cell types, such as dendritic cells, which has been observed in GK5 mice [50]. In support of a role for CD4^+^ T-cells in leukemia control, we have recently shown that these cells are able to control CLL progression in the absence of CD8^+^ T-cells in the TCL1 AT mouse model in an *Eomesodermin* (*Eomes)*- and *Il10rb*-dependent manner [10]. However, it remains unclear whether cytotoxic CD4^+^ T-cells are involved in CLL control in immunocompetent mice with functional CD8^+^ T-cells, and whether such cells are of relevance in CLL patients.

In conclusion, in vitro as well as xenograft experiments suggest that CD4^+^ T-cells enhance CLL cell survival and proliferation, which is supported by correlations of CD4^+^ T-cell counts and clinical outcome. Nevertheless, results of the Eµ-TCL1 mouse model suggest a more diverse role of CD4^+^ T-cells within a complex immune microenvironment. As this mouse model might be limited in its ability to reflect the situation in CLL patients, the use of humanized PDX mouse models containing all major immune cell subsets of human origin might help to clarify whether CD4^+^ T-cells have a pro- or anti-tumoral role in CLL.

## CD8^+^ T-cells

### CD8^+^ T-cell subsets and their role in immune responses

CD8^+^ T-cells are pivotal for adaptive immunity, for example, in response to a viral infection [51]. Activation of CD8^+^ T-cells requires multiple signals that lead to their clonal expansion and differentiation into effector cells. These cells are functionally and phenotypically heterogeneous, and include short-lived effector T-cells (SLEC) that undergo apoptosis once an acute infection is cleared [51], but also effector cells that survive after antigen clearance and form a memory population [52]. CD8^+^ T-cells target infected cells by secretion of perforins, which permeabilize the cell membrane of target cells and enable the diffusion of granzymes into target cells, which cause cell death [53]. In addition, CD8^+^ T-cells can induce clearance of target cells by induction of apoptosis via FAS ligand (FASL) [54].

Long-lived memory CD8^+^ T-cells develop during acute infections from effector cells to provide a fast immunological response upon repeated infections [52]. Effector memory T-cells (T_EM_), which express CD45RO and are negative for C-C motif chemokine receptor 7 (CCR7) in humans, reside in non-lymphoid tissues and retain cytolytic activity [55]. A fraction of T_EM_ expresses CD45RA and is therefore defined as T_EMRA_. These cells contain the highest amounts of perforins [55]. In contrast, CD45RO^+^, CCR7^+^ central memory (T_CM_) cells home to secondary lymphoid organs and are not capable of immediate effector function [55]. A further subset of memory T-cells, CD103^+^ tissue-resident memory T-cells (T_RM_) has been recently identified [56]. T_RM_ cells reside in non-lymphoid tissues, such as the skin. They exert first responses after infections in the tissues but do not recirculate [56].

### CD8^+^ T-cells in conditions of chronic antigen persistence

During chronic infections and in cancer, the persistence of antigens leads to a gradual loss of CD8^+^ T-cell function, a process called T-cell exhaustion [57]. Continuous antigen-driven activation of CD8^+^ T-cells causes the upregulation of multiple inhibitory receptors, such as PD-1, CD244 (2B4), lymphocyte activation gene 3 (LAG-3), and CD160 [57]. In addition to enhanced expression of these inhibitory receptors, exhausted T-cells gradually lose their proliferative potential and the ability to produce cytokines such as IL-2, tumor necrosis factor-alpha (TNFα), and IFNγ [57]. These functional changes are associated with a distinct epigenetic profile that differs from that of effector and naïve T-cells, and cannot be reversed by antibody-mediated immune checkpoint blockade targeting PD-1 [58, 59]. PD-1 blockade has been shown to induce proliferation and reactivation of a precursor exhausted CD8^+^ T-cell subset with features of self-renewal and the ability to differentiate into terminally exhausted T-cells [60].

Besides in chronic viral infections, for example with human immunodeficiency virus (HIV), CD8^+^ T-cell exhaustion has been described in several cancer entities, including non-small cell lung carcinoma (NSCLC) and melanoma [61–63].

### CD8^+^ T-cells in CLL

Elevated T-cell numbers in the blood of CLL patients were described as long as 40 years ago [42, 43, 64, 65] with the observation of the decrease of the CD4 : CD8 T-cell ratio coming shortly after [17, 42, 49, 66–70]. An inversion of the CD4 : CD8 T-cell ratio with values below 1 was found to be associated with shorter time to first treatment (TTFT), shorter overall survival (OS) [6, 71], and shorter PFS independently of other prognostic markers [6, 11]. Using a ratio of CD8^+^ T-cells per monoclonal, malignant B-cell in CLL blood samples, higher relative numbers of CD8^+^ T-cells correlated with better OS [72], suggesting that CD8^+^ T-cells might participate in leukemia control.

In addition to the altered numbers of CD8^+^ T-cells, detailed descriptions of their phenotype and leukemia-associated changes in blood of CLL patients are also available. The CD8^+^ T-cells of CLL patients were found to be enriched in antigen-experienced effector memory and effector cells [6–9, 11, 14, 43, 49, 70, 73–75] and were shown to express markers of T-cell activation such as HLA-DR and CD69 more strongly than cells of healthy individuals [6, 42, 49]. Unsupervised computational analysis of the expression levels of 29 proteins analyzed by flow cytometry showed that blood-derived CD8^+^ T-cells from CLL patients are phenotypically distinct from those of healthy donors [76].

In line with the concept of T-cell exhaustion as an activation-induced dysfunction, several reports have independently demonstrated increased expression of exhaustion markers. Initiated by Motta et al. [40], and followed by Nunes et al. [11] and Riches et al. [9], higher expression of PD-1 [9, 11, 49, 71, 74, 77, 78], CD160 [9], CD244 [9], T-cell immunoglobulin mucin 3 (TIM-3) [78], killer cell lectin-like receptor G1 (KLRG1) [75] and CTLA-4 [7, 40] was reported on CD8^+^ T-cells in the blood of CLL patients. Counts of PD-1^+^ CD8^+^ T-cells were found to positively correlate with CLL burden [7, 49]. These results were recapitulated in the Eµ-TCL1 and TCL1 AT mouse models of CLL, implying that these models are useful to investigate CD8^+^ T-cell immunity in CLL [14, 17, 38, 39, 49, 79, 80].

#### Function or dysfunction of CD8^+^ T-cells in CLL

Although detailed phenotypic characterizations of CD8^+^ T-cells in CLL are available, investigations of their role during development and progression of CLL are largely missing. The idea of defective or diminished leukemia control by CD8^+^ T-cells was supported by the reduced proliferative capacity and target cell lysis observed in patient blood-derived CD8^+^ T-cells after T-cell receptor (TCR) engagement ex vivo [9]. Moreover, gene expression analysis of patient-derived CD8^+^ T-cells revealed deregulation of actin polymerization, vesicle formation and trafficking, and cytotoxicity-associated pathways, in comparison to control T-cells [12]. Further, CD4^+^ and CD8^+^ T-cells of CLL patients were found to form impaired immunological synapses with CLL cells. Super-antigen pulsed CLL cells from untreated patients formed less conjugates with autologous T-cells, and a lower F-actin accumulation at the immune synapse was detected in comparison to experiments with healthy donor B- and T-cells [81], which was also seen in a study using T-cells from Eµ-TCL1 mice [37]. Impaired immune synapse formation was further observed when healthy donor-derived T-cells were co-cultured with CLL cells for 48 h, suggesting that this defect is induced by the malignant CLL cells. Blocking inhibitory ligands, such as CD200, programmed cell death 1 ligand 1 (PD-L1), or CD276, with neutralizing antibodies in CLL co-cultures improved this defect of immune synapse formation [82].

In the recent years, several reports suggested an active anti-leukemia immunity by CD8^+^ T-cells, as TCR analyses of CLL blood samples or spleens of TCL1 AT mice revealed an enrichment of clonally expanded CD8^+^ T-cells [49, 83–85] with the major clonotypes persisting for about 2 years in patients [83]. This concept is further supported by an analysis of HLA ligandomes, in which CLL-specific antigens, which cause spontaneous, autologous T-cell activation, were identified [86]. In addition, we recently reported that CD8^+^ T-cells are able to control CLL progression in the TCL1 AT model which leads to longer survival of leukemic mice in the presence of these immune cells [49]. This is in line with a recent study that analyzed CLL development in Eµ-TCL1 mice crossed with mice that harbor a B-cell specific knockout of interferon regulatory factor 4 (IRF4) [87]. Lack of IRF4 in malignant B-cells resulted in faster development of CLL, which was associated with a downregulation of major histocompatibility complex (MHC) molecules on CLL cells and reduced activation of T-cells, suggesting an abrogation of T-cell-mediated leukemia control in these mice [87].

#### Exhaustion of CD8^+^ T-cells in CLL

**Fig. 2:**
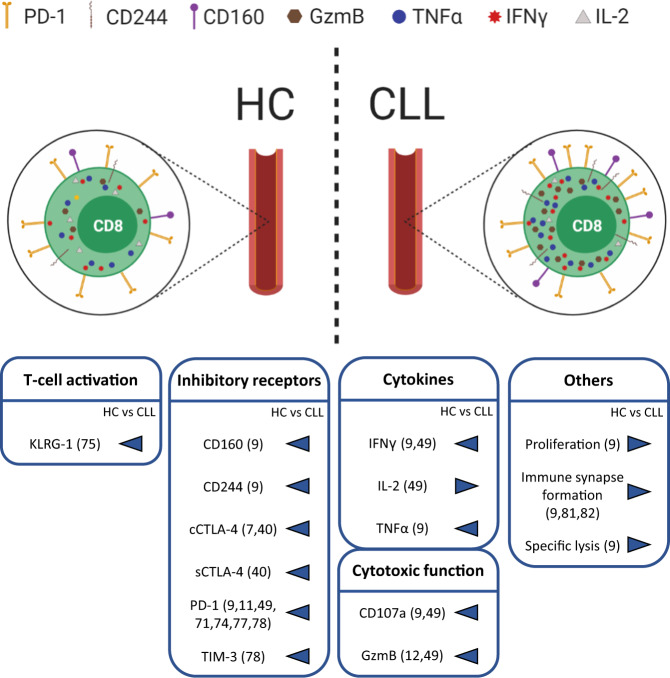
Comparison of CD8^+^ T-cells from blood of CLL patients and healthy controls. Blood-derived CD8^+^ T-cells in CLL harbor a higher expression of inhibitory receptors PD-1, CD244, and CD160, and of effector molecules granzyme B (GzmB), TNFα, IFNγ and IL-2 compared with respective cells of healthy controls (HC). Overview of differential marker expression of T-cell activation, inhibitory receptors, cytokines and cytotoxic molecules as well as other T-cell functions of CD8^+^ T-cells from HC (left) versus CLL patients (right). cCTLA-4: cytoplasmatic expression of CTLA-4; sCTLA-4: surface expression of CTLA-4.

**Fig. 3:**
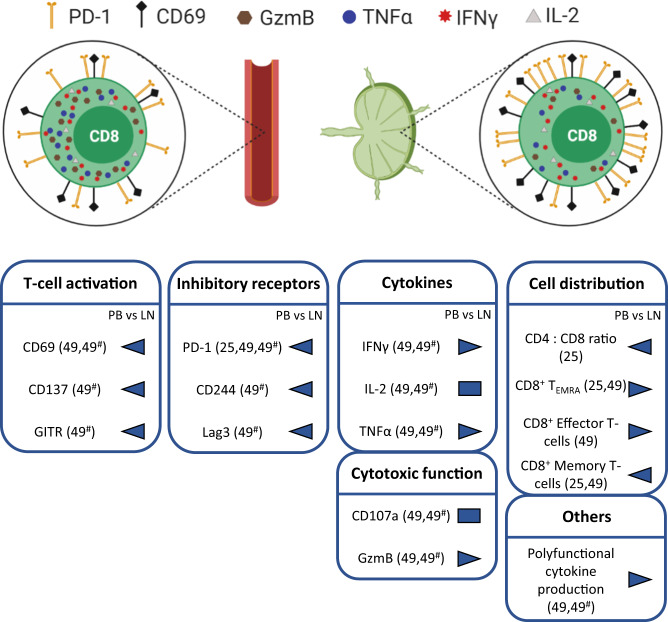
T-cell exhaustion phenotype of CD8^+^ T-cells in blood versus lymph nodes of CLL patients. Comparison of blood (left) and lymph node-derived (right) CD8^+^ T-cells of CLL patients. CD8^+^ T-cell exhaustion is more severe in lymph nodes compared with blood of CLL patients, with increased expression of PD-1 and CD69, as well as reduced production of effector molecules. Overview of differential marker expression of T-cell activation, inhibitory receptors, cytokines and cytotoxic molecules, cell distribution and other CD8^+^ T-cell functions of blood (PB, left) versus lymph nodes of CLL patients (LN, right) or spleens of CLL mouse models. T_EMRA_: effector memory RA T-cells; ^#^ results derived from the TCL1 AT mouse model of CLL.

As mentioned above, a hallmark of T-cell exhaustion is enhanced expression of inhibitory receptors, such as PD-1, along with decreased cytokine production after ex vivo stimulation [88]. Although CLL patient blood-derived CD8^+^ T-cells have been shown to express PD-1 and other inhibitory receptors, accompanied by defective immune synapse formation [9], increased cytokine production by these cells has also been described (Fig. 2) [9, 49]. This has resulted in the conclusion that CD8^+^ T-cells in CLL are “pseudoexhausted” [9]. Building on these findings, a comparative analysis of CD8^+^ T-cells of paired blood and lymph node samples of CLL patients showed an accumulation of PD-1^+^ CD8^+^ T-cells harboring all key features of exhaustion in lymph nodes but not blood samples, namely the expression of several inhibitory receptors and reduced cytokine production (Fig. 3) [25, 49]. The presence of such exhausted T-cells was confirmed in spleen samples but to a much lower extent in blood of TCL1 AT mice [49], suggesting that T-cell exhaustion in CLL is a phenomenon that happens in lymphoid tissues rather than blood.

In conclusion, emerging data clearly emphasize that CD8^+^ T-cells in CLL likely recognize tumor-specific antigens, but fail to control disease due to their functional exhaustion, a phenotype that is more pronounced in lymphoid organs compared with the blood of CLL patients.

## Impact of novel kinase inhibitors on T-cells

### PI3Kδ inhibitors impair T-cell function in CLL

Inhibition of phosphatidylinositol 3-kinase δ (PI3Kδ) by idelalisib has proven to be a clinically efficient treatment option for CLL [89–91]. However, immune-related adverse events were observed during this treatment, such as autoimmune-mediated elevation of liver enzymes associated with an infiltration of lymphocytes in the liver, as well as a decrease in Tregs [92], and increased rates of infections [89–91, 93, 94]. As PI3Kδ is expressed by all leukocytes [95], T-cells are also affected during treatment with PI3Kδ inhibitors which contributes to such adverse events [32, 96, 97]. Idelalisib treatment of CLL patients resulted in reduced numbers, proliferation, and suppressive function of Tregs, which was confirmed in the TCL1 AT mouse model [32, 92, 96, 97]. Ex vivo analysis of T-cells from idelalisib-treated patients before and during treatment revealed a reduced expression of activation markers in Tregs in treated samples [97]. Reduced Treg activity by PI3Kδ inhibition was shown to unleash CD8^+^ T-cell activity, leading to better CD8^+^ T-cell-mediated control of solid tumors [98]. In contrast, PI3Kδ inhibitor treatment of leukemic TCL1 AT mice resulted in reduced differentiation of CD8^+^ T-cells towards antigen-experienced effector cells and diminished effector function [32], which was likely caused by diminished TCR signaling by PI3Kδ inhibition [32, 97].

In conclusion, clinically approved PI3Kδ inhibitors can cause a potentially beneficial reduction of immunosuppressive functions of Tregs. However, these drugs were also shown to reduce CD8^+^ T-cell effector capacity. Therefore, the imbalance of regulatory and cytotoxic T-cell subsets likely explains autoimmune events as well as increased infection rates in idelalisib-treated CLL patients.

### Ibrutinib inhibits TCR signaling in CLL

Ibrutinib, an irreversible Bruton’s tyrosine kinase (BTK) inhibitor commonly used for the treatment of CLL, was shown to inhibit interleukin-2-inducible T-cell kinase (ITK), which is important for the activity of T-cells [99], as reviewed recently by Mhibik et al. [100]. Analysis of T-cells derived from ibrutinib-treated CLL patients revealed an occupancy of ITK by ibrutinib of up to 55% [99]. Furthermore, a decline in total T-cell numbers as well as CD4^+^ and CD8^+^ T-cell numbers in peripheral blood was reported during treatment with ibrutinib [101, 102]. In contrast, Long et al. noted an increase in T-cell numbers in a cohort of 18 CLL patients treated with ibrutinib [103]. Besides its impact on T-cell numbers, ibrutinib was shown to reduce the activation, proliferation, and cytokine production of T-cells upon treatment ex vivo [104] and in the TCL1 AT mouse model of CLL [105]. Moreover, ibrutinib but not acalabrutinib, a covalent BTK inhibitor with lower affinity for ITK, reduced TCR signaling of murine T-cells in vitro [105]. Other recent data have further shown that CLL patients gained a broader TCR repertoire diversity after 1 year of treatment with ibrutinib compared with pre-treatment samples [101].

As ibrutinib leads to an efficient reduction of CLL cells in almost all affected tissues, the described alterations within the microenvironment of ibrutinib-treated patients might be due to a diminished impact of the malignant cells on their surroundings. Therefore, it still remains to be elucidated whether ibrutinib-mediated alterations of the T-cell compartment in CLL patients are due to a direct inhibition of ITK in T-cells, or indirectly due to the reduction of CLL tumor burden by ibrutinib and a subsequent normalization of the inflammatory milieu [101–103, 106–109].

In summary, ibrutinib blocks ITK, a kinase that regulates T-cell activation after TCR engagement, which could explain reduced numbers of T-cells with a more diverse TCR repertoire in ibrutinib-treated CLL patients. However, ongoing studies with acalabrutinib and other novel and more specific BTK inhibitors will ultimately clarify whether the reported effects are due to ITK inhibition or normalization of the inflammatory microenvironment in ibrutinib-treated CLL patients.

## Immunotherapeutic approaches involving T-cells

### Immune checkpoint blockade

Immune checkpoint blockade has changed the treatment strategies of many different cancer entities. The disruption of the interaction of PD-1 and its ligands and the blocking of CTLA-4 are the best-studied examples.

In CLL, initial pre-clinical investigations were encouraging. Using a blocking antibody against PD-L1 in the TCL1 AT mouse model of CLL achieved a substantial reduction of leukemia burden and a reversion of the exhausted T-cell phenotype [110]. Comparable results were obtained combining antibody therapies directed against PD-1 and LAG-3 [38]. Of note, single-agent treatment with αPD-1 antibodies did not reduce leukemia development in this study [38]. In line with this, treatment of 16 relapsed CLL patients with a humanized αPD-1 antibody also did not result in a response in any of the CLL patients [111]. However, four out of nine patients harboring Richter transformation showed a response to this treatment (NCT02332980) [111]. As these results were disappointing, combination therapies of kinase inhibitors and checkpoint blockade are currently under investigation (NCT02329847, NCT02332980, NCT02362035, NCT02420912, NCT03514017, NCT03153202, and NCT03283137) and first results indicate response rates of 61% [112]. This agrees with encouraging results in the TCL1 AT model, where combination of ibrutinib with either αPD-1 or αPD-L1 considerably improved leukemia control and CD8^+^ T-cell effector function, with more pronounced effects in the ibrutinib/αPD-L1 combination [105]. Of interest, this combination led to an almost complete eradication of CLL cells in the bone marrow, which was not achieved with ibrutinib or checkpoint blockade monotherapies, suggesting that the combination of ibrutinib with αPD-L1 might be able to eradicate CLL cells more efficiently also in lymphoid tissues [105].

In conclusion, more investigations are needed to explore the potential of immune checkpoint blockade in CLL and to develop rational combination approaches to overcome T-cell dysfunction and immune escape in this disease.

### Chimeric antigen receptor (CAR) cell therapies

Increasing the specificity of immune cells against cancer cells by genetic introduction of a CAR has boosted immunotherapeutic approaches in B-cell leukemia and lymphoma. Thus far, two products, tisagenlecleucel and axicabtagen-ciloleucel, have been approved by the Food and Drug Administration (FDA). About 60% of CLL patients treated within clinical trials with autologous CAR T-cells directed against the B-cell antigen CD19, showed a response [113, 114]. Complete remissions were seen in about 29% [113], and 21% [114] of CLL patients treated with CAR T-cells, which is considerably lower than in B-cell acute lymphoblastic leukemia patients. Estimated median progression-free survival of CD19 CAR T-cell treated CLL patients was reported as 7 months [113] or 8.5 months [114]. During the treatment with CAR T-cells, a high rate of patients suffered of side effects such as cytokine release syndrome [113, 114].

Major limitations of the CAR T-cell approach were recently overcome using cord blood-derived, CAR-transduced natural killer (NK)-cells (NCT03056339) [115]. The advantage of this approach is that no full HLA match between donor and host cells is required, which circumvents the necessity of using autologous cells for CAR transduction, thereby shortening and simplifying the generation of CAR cell products [115]. CAR NK-cell treatment induced remission in four of five patients with CLL, including patients harboring Richter transformation [115].

In summary, more therapeutic options that are based on T-cells and other immune cells, are currently being developed for CLL which have the potential to complement the small molecule-based treatment paradigms of CLL.

## Conclusions and future challenges

This review compiles current knowledge of CLL-associated alterations in T-cell distribution, phenotypes, and function. Although it is now well established that CLL disease development elicits accumulation of CD4^+^ and CD8^+^ T-cells, the role of these cells in either supporting the growth of malignant B-cells or controlling disease progression by adaptive immune responses is still under debate. The observed phenotype and functional properties of CD8^+^ T-cells in CLL, along with their oligoclonal expansion, suggest an ongoing CLL-reactive adaptive immunity. Failure of tumor control in CLL is likely due to activation-induced exhaustion of T-cells, as described in other cancers. Of note, the exhaustion phenotype of CD8^+^ T-cells in CLL was shown to be more severe in lymphoid organs compared with blood, which is likely explained by the tight interactions of T-cells and CLL cells in pseudofollicles in these tissues, leading to immune cell activation. Therefore, future work investigating T-cells in CLL should aim at verifying results derived from blood samples of CLL patients by analyses of cells from lymphoid organs, mouse models, or tissue culture models that mimic the complex lymphoid microenvironment in CLL.

Novel targeted therapies for CLL, such as the inhibition of BTK or PI3Kδ not only impact on the malignant B-cells, but also target other immune cells in the microenvironment of CLL, including T-cells. To overcome adverse events, it is vital that the impact of such inhibitors on immune cell function, particularly T-cell activity, is monitored closely during clinical trials. This will not only increase the safety for patients but also result in a better understanding of the immune compartment under different treatment regimens, which will be essential for the development of rational combination therapies, including immunomodulating agents.

With the availability of novel technologies, such as multiplexed single-cell protein analysis by mass cytometry or single-cell transcriptome analyses, it has become clear that immune cells are much more heterogeneous than initially thought. One example is the stepwise activation and exhaustion process of CD8^+^ effector T-cells, where the presence or absence of distinct intermediate cell stages might have profound effects in terms of response to immunotherapies. Therefore, in-depth analyses of T-cells in cancer patients using these novel techniques will be essential to predict outcome and therapy response. In addition, such studies will help to understand processes of immune cell activity and dysfunction and will, therefore, be essential for the identification of novel therapeutic targets. In light of the disappointing treatment results with immune checkpoint inhibitors and CAR T-cell approaches in CLL, it has become clear that our knowledge about the T-cell compartment in this disease is unsatisfactory, and we must strive to deepen our understanding of immune control and escape in CLL in the future.
