# Publisher Correction: T cells in chronic lymphocytic leukemia: guardians or drivers of disease?

**DOI:** 10.1038/s41375-021-01362-7

**Published:** 2021-11-16

**Authors:** Philipp M. Roessner, Martina Seiffert

**Affiliations:** Molecular Genetics, German Cancer Research Center (DKFZ), Heidelberg, Germany

**Keywords:** Cancer microenvironment, Chronic lymphocytic leukaemia

Correction to: *Leukemia* 10.1038/s41375-020-0873-2

The article T cells in chronic lymphocytic leukemia: guardians or drivers of disease?, written by PMR and MS, was originally published Online First without Open Access. After publication in volume 34, page 2012–2024 the author decided to opt for Open Choice and to make the article an Open Access publication. Therefore, the copyright of the article has been changed to © The Author(s) 2020 and the article is forthwith distributed under the terms of the Creative Commons Attribution

